# Periodontal infectogenomics: a systematic review update of associations between host genetic variants and subgingival microbial detection

**DOI:** 10.1007/s00784-021-04233-8

**Published:** 2022-02-05

**Authors:** Noha Zoheir, Yuko Kurushima, Guo-Hao Lin, Luigi Nibali

**Affiliations:** 1 Periodontology Unit, Centre for Host-Microbiome Interactions, Dental Institute, King’s College London, Great Maze Pond, London, UK; 2 University of California, San Francisco, CA USA

**Keywords:** Genetic, Bacteria, Periodontitis, Infectogenomics

## Abstract

**Objective:**

The aim of this study was to systematically update the evidence for associations between host genetic variants and subgingival microbial detection and counts.

**Materials and methods:**

Following a previous systematic review (Nibali et al. J Clin Periodontol 43(11): 889-900, 15), an update of a systematic search of the literature was conducted in Ovid Medline, Embase, LILACS, and Cochrane Library for studies reporting data on host genetic variants and detection of microbes subgingivally published in the last 6 years.

**Results:**

A total of 19 studies were included in the review, from an initial search of 2797 titles. Studies consisted mainly of candidate gene studies and of one genome-wide analysis. A total of 62 studies were considered for summary findings, including 43 identified in the previous systematic review of studies published up to 2015. Meta-analyses were done when appropriate including both papers in the original review and in the update. Meta-analyses revealed lack of associations between *IL1* composite genotype and subgingival detection of *Aggregatibacter acinomycetemcomitans*, *Poprhyromonas gingivalis*, *Tannerella forsythia*, *Treponema denticola*, and *Prevotella intermedia*. Promising evidence is emerging from other genetic variants and from sub-analyses of data from genome-association studies. Among other studies with candidate-gene, target SNPs were mainly within the *IL10*, *IL6*, *IL4*, *IL8*, *IL17A*, and *VDR* gene.

**Conclusions:**

*IL1* composite genotype does not seem to be associated with subgingival microbial detection. Promising associations should be pursued by future studies, including studies employing -OMICS technologies.

**Clinical relevance:**

A better knowledge of which host genetic variant predispose to subgingival microbial colonization and to the development of progression of periodontal disease could potentially help to better understand periodontal disease pathogenesis and help with its management.

**Supplementary Information:**

The online version contains supplementary material available at 10.1007/s00784-021-04233-8.

## Background

Humans are considered ‘holobionts’ who have evolved with their colonizing microbes. A large part of the human microbiota is vital for health and survival, although some microbes may have harmful effects and predispose to human disease [1]. Periodontitis is a microbial dysbiosis-initiated inflammatory disease of the supporting apparatus of the teeth. A multitude of factors such as systemic, environmental, and genetic may directly or indirectly influence disease initiation and progression at multiple levels [2, 3]. Genetic factors have been strongly associated with periodontitis [4]. The effects of these factors have been extensively studied over the last decades, resulting in a significant paradigm shift in the etiology of periodontal disease. A myriad of host factors is potentially responsible for the composition of the oral microbiome and therefore for affecting disease susceptibility [5]. There is now an increased emphasis on genetic variants as modifiers of microbial dysbiosis and of associated diseases [6, 7].

‘Infectogenomics’ has been introduced as a term to define the effect of host genetic variants (namely single-nucleotide polymorphisms, or SNPs) in influencing the response to infective agents and therefore the risk to develop disease [8]. Dysbiotic diseases such as periodontitis may also be influenced by the effect of host genetic variants [9]. Specific subgingival bacteria seem to be affected by some host genetic variants, as shown in candidate gene analysis as well as genome-wide association studies (GWAS) [10–14]. A better knowledge of which host genetic variant predispose to microbial colonization below the gingival margin and to the development of progression of periodontal disease could potentially help the understanding of periodontal disease pathogenesis and help with its management. Therefore, it is important to assess these potential associations systematically. A previous systematic review of these associations [15] showed a lack of evidence to support that host genetic polymorphisms are associated with presence and counts of subgingival bacteria. It was also suggested that further studies on large populations with replication samples should clarify the possible effects of other genetic variants on the subgingival microbiota were conducted. Following that review, several original studies proving more evidence were published. Therefore, a systematic review update, with new discussion and analysis including original data produced in the last 6 years was carried out.

## Materials and methods

A systematic review protocol was written in the planning stages and the PRISMA checklist [16] was followed both in planning and reporting this review (checklist attached as supplemental material 1). A review protocol was prepared and registered with PROSPERO (reference CRD42020190636).

Broad question:

- What is the association between host genetic variants and detection of specific microbes subgingivally?

PECOS outline:

- Population: subjects with measures of periodontal disease or periodontal health
- Exposure: analysis of host genetic variants
- Comparisons: genotypes/allele frequency at different SNPs
- Outcomes: detection of specific microbes subgingivally
- Studies: case–control, cross-sectional, cohort or randomized controlled trials (RCTs)

### Information sources

Following a previous systematic review [15], the search was conducted through the electronic databases MEDLINE, EMBASE, LILACS, and The Cochrane Database [including the Central Register of Controlled Trials (CENTRAL)] and was complemented by a search through the reference lists of included studies. No language restriction was included in the initial search. Among published literature, peer-reviewed studies, reports, book chapters, and conference abstracts were screened. Narrative or systematic reviews on the topic were searched in order to identify suitable papers.

### Search strategy

The search strategy used a combination of MeSH terms and key words described in supplemental material 2. Papers published between 11^th^ September 2015 (after the close date of the previous review) and 30^th^ May 2021 were searched.

### Study selection-eligibility criteria

Human studies reporting measures of associations between host genetic variants and detection of subgingival microbes were considered suitable for this review. Inclusion criteria were:

- Study designs:

- Case–control studies
- Cross-sectional studies
- Longitudinal/cohort studies or RCTs providing baseline genetic and microbial data

- Reporting measures of periodontal disease reported (periodontal diagnosis)
- Reporting analysis of host genetic variants (SNPs)
- Reporting data on microbial detection subgingivally (by host genetic variant)

Exclusion criteria were:

- Reviews
- Case reports
- Studies on animal models

Study selection was conducted by two independent reviewers (authors NZ, YK) in the following stages:

1. Initial screening of potentially suitable titles and abstracts against the inclusion criteria to identify potentially relevant papers
2. Screening of the full papers identified as possibly relevant in the initial screening

Studies were excluded if not meeting the inclusion criteria (such as for instance animal studies, conference abstracts, or reviews). Following the screening of titles and abstracts (steps 1 and 2), the studies included by both reviewers were compared and a complete database for step 3 was formed joining all studies selected by at least one reviewer. Following step 3, in case of a disagreement between reviewers, the decision about study eligibility was made trying to reach a consensus between the two reviewers. In case of continued disagreement, a third reviewer or arbitrator (author LN) judged study inclusion. The agreement value between reviewers will be calculated after step 2 and after step 3 using Kappa statistics.

### Data collection process/data items

Data were extracted based on the general study characteristics (authors and year of publication, country, and study design) and population characteristics (number of participants, age, gender, ethnicity, inclusion/exclusion criteria, and diagnosis of periodontal status). Specific data on genetic and microbial analysis, genetic variants analyzed, microbes analyzed, method used for genetic analysis, and method used for microbial sampling and microbial detection/identification were extracted, as previously described [15].

### Risk of bias in individual studies

The risk of bias of the included case–control and cross-sectional studies was assessed through sensitivity analysis by using a recently proposed score of 0 to 20 adapted to genetic analyses of periodontal studies [17]. The ‘Newcastle Ottawa tool to assess risk of bias’(Newcastle Ottawa scale http://www.ohri.ca/programs/clinical_epidemiology/oxford.htm) was used to assess risk of bias for longitudinal studies.

### Summary measures/synthesis of results/statistical methods

The study outcomes were the risk ratio of detection of specific subgingival microbes (primary outcome) or the overall microbial counts or proportions (secondary outcome) in patients with different genotypes. We aimed to stratify results separately according to periodontal diagnosis (periodontitis, gingivitis, health) if possible. The studies identified in the current updated review were pooled with the 43 studies identified in the original review [15] to assess for possible meta-analysis. A meta-analysis was considered appropriate and performed in the presence of a significant number of similar studies addressing the same question (and analyzing the same gene variants and subgingival microbes) and judged of acceptable quality [18].

The study outcomes were the risk ratio of detection of specific subgingival microbes (primary outcome) or the overall microbial counts or proportions (secondary outcome) in patients with different genotypes. Meta-analysis could be performed in the presence of at least 3 papers investigating the same combination of SNPs and subgingival bacteria. The risk ratios of primary and secondary outcomes were estimated using a computer program (Review Manager Version 5.0. Copenhagen; The Nordic Cochrane Centre, The Cochrane Collaboration, 2008). The contribution of the included articles was weighted using inverse-variance method. Random effects meta-analyses of the selected studies were applied if the heterogeneity is considered moderate to high among the pooled studies; otherwise, fixed effects meta-analyses were applied if the heterogeneity is low. Forest plots were produced to graphically show the difference in outcomes of groups with different genotypes using number of SNP with each genotype as the analysis unit. A *p* value = 0.05 was used as the cut-off level for significance. Heterogeneity was assessed with chi-square tests and *I*^2^ test, which ranges between 0 and 100% and where lower values represent less heterogeneity.

## Results

### Study selection

**Fig. 1:**
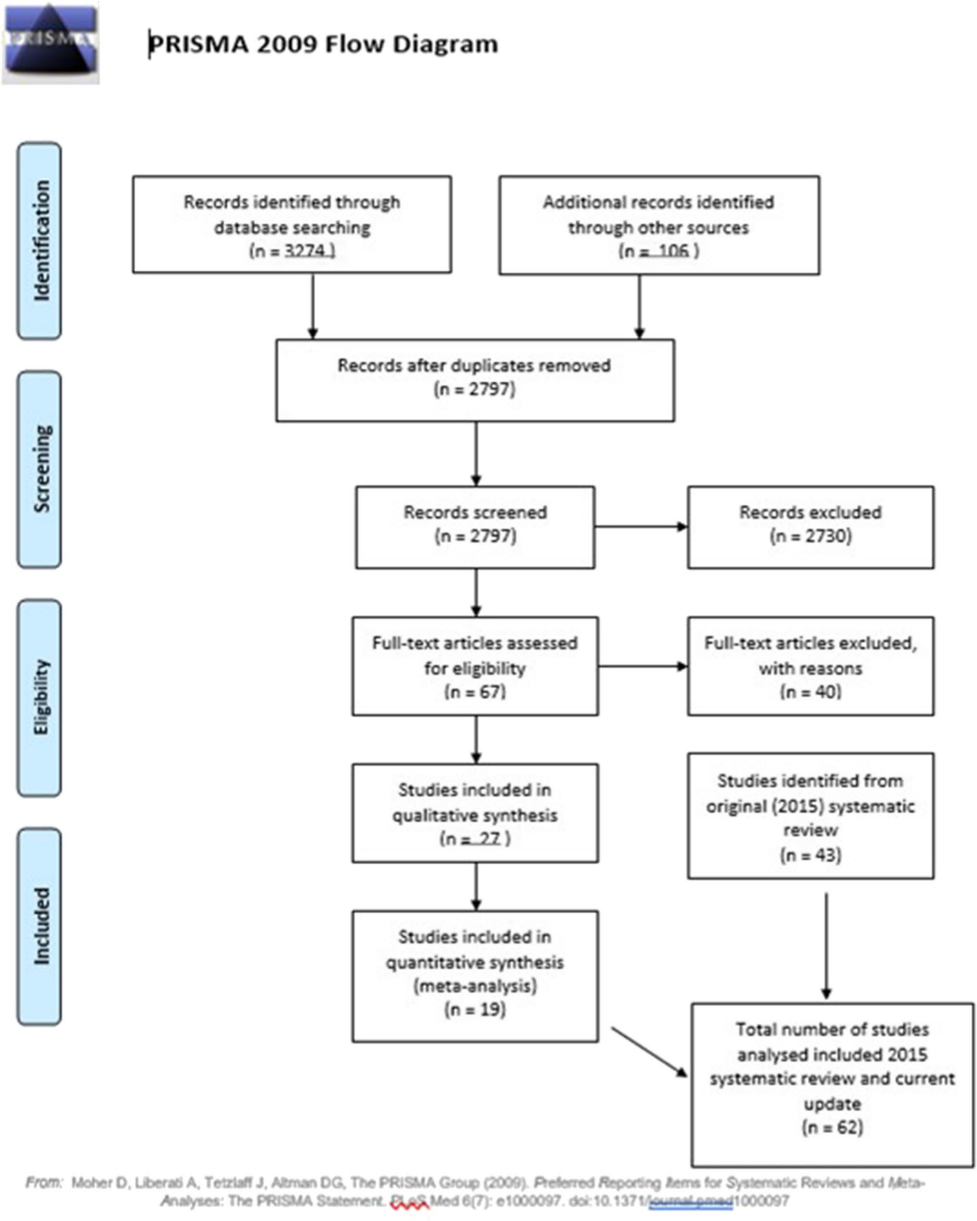
Flowchart of study inclusion

Figure 1 shows the flowchart representing study selection and inclusion. The initial search resulted in 2797 papers published between 11^th^ September 2015 (after the close date of the previous review) and 30^th^ May 2021 were searched at Ovid Medline, Embase, Cochrane Library, and LILACS combined. Following first-stage screening of titles and abstracts, 70 articles qualified for full-text screening (considered potentially suitable by at least one reviewer). After full text reading, 19 articles met the defined inclusion criteria and 51 were excluded (see Fig. 1 for reasons for exclusion). The kappa value for inter-reviewer agreement was 0.41 at title and abstract screening and 0.80 at full text reading.

### Study characteristics

Table 1 reports the characteristics of the reviewed studies. All of the 19 included studies were written in English. The countries where the studies were conducted included Brazil (*n* = 4), USA (*n* = 5), Czech Republic (*n* = 3), China (*n* = 2), Italy (*n* = 2), Spain (*n* = 1), Thailand (*n* = 1), and Macedonia (*n* = 1). The number of study participants ranged from 39 to 4910. The majority of studies had a case–control design in a University setting, while only 1 study was a longitudinal treatment study.

**Table 1:** Summary of study characteristics and of genetic and microbiological methods and main findings for 19 studies included from our literature search and update in this review

Authors	Study design	Ethnicity	Number of patients	Clinical diagnosis	Genetic analysis -Method -Analyzed gene	Microbiological analysis -Method -Analyzed bacteria	Associations-main infectogenomics results
Torungruanget al. 2020	CS	Thai	1460	CP, H (smokers included)	RT-PCR VDR FokI	RT PCR Aa, Pg, Tf, Td, Pi	FokI risk genotypes (CC + CT)—Pg
Karikova 2019	CC	Caucasian	500	CP, H (smokers included)	PCR CXCR2 (SNPs): + 785C/T, + 1208 T/C. + 1440A/G	DNA microarray Aa, Tf, Pg, Td, Parvimonas micra, Pi, Fn	NS
Cavalla 2018 [34]	CC	Brazilian	167	CP, H	qPCR Genome-wide SNP array	DNA-DNA hybridization 40 species	rs10010758, rs6667202: increased counts of Pg; rs10043775: decreased counts of Pi; rs2521634: decreased counts of Tf, Ag, Fp, and Pn
Geng 2018 [32]	CS	Chinese	266	CP, AgP, H	PCR and Snapshot Multiplex IL-10–592, -819 and-1082 SNPs	RT PCR Pg, Aa	IL-10 ATA/ATA genotype: increased Aa (IL-10–819 TT-higher Aa than TC)
Mesa 2017 [24]	CS	Caucasian	203	CP, AgP, H (smokers included)	Multiplex PCR IL-1A, IL-1B and IL-1RN	PCR and reverse hybridization Pg, Aa, Tf, Td, Pi	IL-1RN polymorphism: lower counts of Pg, Tf and Pi
Marchesan 2017 [22]	CS	Caucasian	4766	Cross-sectional (ARIC study) (smokers included)	Bioinformatics IFI16, AIM2	DNA chromosomal checkerboard array Pg, Aa, Tf, Td, Pi, Cr, Fn, Pn	rs6940 and rs1057028: increased Pg, Tf and Cr Haplotype block rs1057028: association with Fn and Aa
Lauritano et al. 2016 [31]		Caucasian	326	CP (smokers not excluded)	PCR VDR, IL-6, IL-10	Reverse hybridization Pg, Aa, Tf, Td, Cr, Fn	NS
Offenbacher et al. 2016 [5]	CS	Caucasian	975	Cross-sectional (ARIC study) (smokers included)	Affymetrix Genome-Wide Human SNP Array 6.0 chip -Different SNPs linked to PCT 1 to 6	Checkerboard array Pg, Aa, Tf, Td, Pi, Cr, Fn, Pn	PCT1 was characterized by a uniformly high pathogen load, whereas PCT3 and PCT5 were dominated by Aa and Pg respectively
Sellers et al. 2016 [21]	CC	Ns	617	CP, H, RA and osteoarthritis (smokers included)	Immunichip assay TLR4 Asp299Gly and CD14	Reverse hybridization Pg	Negative interaction between the TLR4 SNP and Pg
Linhartova et al. 2015	CC	Caucasian	469	CP, H (smokers included)	RT PCR ApoE	Pathogen detection kit Pg, Aa, Tf, Td, Pi, Pm, Fn	NS
Stojanovska et al. 2019	CC	Macedonian	40	CP, H (smokers included)	PCR IL-1a and IL-1B	Reverse hybridization Pg, Aa, Tf, Td, Pi	NS
Cavalla et al. 2015 [14]	CC	Brazilian	175	CP, H (smokers included)	PCR–RFLP IL-10	RT PCR Pg, Aa, Tf, Td	NS
Cirelli et al. 2017 [37]	CC	Brazilian	104	CP, H (smokers included)	PCR–RFLP IL-4 and IL-8	RT PCR Pg, Aa, Tf, Td	IL4 haplotypes: high levels of Aa before and after perio treatment. After treatment, higher levels of Aa were found in subgingival sites of (IL4–) patients
Mehlotra et al. 2016 [19]	CS	Caucasian + Afro-american	115	CP, HIV + ve (smokers included)	Illumina’s GoldenGate genotyping assay system combined with VeraCode Technology and RT-PCR TLR genes and in DEFB4/103A	RT PCR Pg, Tf, Td	8 SNPs in TLR were significantly associated (Pg, Td, Tf)
Cavalla et al. 2018 [39]	CC	Brazilian	699	CP, AgP, H	PCR CCR5Δ32	RT PCR Pg, Tf, Td	NS
Linhartova et al. 2016 [20]	CC	Caucasian	523	CP, H, T1DM (smokers included)	PCR IL-17	DNA microarray Aa, Tf, Td, Pi, Fn	IL-17A increased Tf and Td in patients with CP and T1DM + CP, respectively
Inchingolo et al. 2020 [36]	CC	Caucasian	96	CP (smokers included)	PCR IL-10, TNFα, IL-1α; IL-1β, IL-1RN,VDRs:Taql, Bsml, Fokl, and COLIA1 (collagen type-l α)	RT PCR Aa, Pg, Tf, Td, Synergistetes, Fl, Micromomes; Fn, Cr, Pi, Rothia, E.c., Ch	positive association: IL-10 genotypes and the presence of Tf, Rd, Ch, Pg, Td, Pm, Synergistetes, Ec

*CC* case–control studies where periodontitis cases where compared with healthy controls; *CS* cross-sectional studies of periodontitis cases or general population, without presence of pre-selected controls; *CP* chronic periodontitis; *AgP* aggressive periodontitis; *H* healthy; *PD* periodontitis; *RA* rheumatoid arthritis; *T2DM* type 2 diabetes mellitus; *SNP* single-nucleotide polymorphism; *PCT* periodontal complex traits; *ARIC* atherosclerosis risk in communities; *NS* no significant associations detected; *Aa Aggregatibacter acinomycetemcomitans*, *Ag Actinomyces gerencseriae*, *Cr Campylobacter rectus*, *Ch Cardiobacterium hominis*, *Ec Eikenella corrodens*, *Fn Fusobacterium nucleatum*, *Fp Fusobacterium periodonticum*, *Fl Fusobacterium lifacto*, *Pm Peptostreptococcus micros*, *Pg Porphyromonas gingivalis*, *Pi Prevotella intermedia*, *Pn Prevotella nigrescens*, *Pe Porhyromonas endodontalis*, *Td Treponema denticola*, *Tf Tannerella forsythia*, *TLR Toll like receptor*, *VDR vitamin D receptors*, *IFI16 interferon γ-inducible protein 16*, *AIM2 absent in melanoma 2*

Included cases ranged from chronic periodontitis (CP), aggressive periodontitis (AgP), gingivitis, and healthy periodontia. Some papers focused only on patients with specific medical history, such as HIV [19], diabetes [20], and rheumatoid arthritis [21]. Two papers described analyses of a large explorative host genome dataset [5, 22], while all other studies focused on a candidate gene with one or a few selected SNPs. Genetic analysis was generally performed by polymerase chain reaction (PCR) after DNA extraction from blood samples (leukocytes) or buccal swabs, with some studies using a chair-side PST (Periodontal Susceptibility Test). Microbiological analysis was generally performed by PCR (see Table 1 for details). Microbial outcomes included detection (presence/absence) or counts or proportions of bacteria. Target bacteria usually consisted of *Aggregatibacter acinomycetemcomitans*, *Poprhyromonas gingivalis*, *Tannerella forsythia*, *Treponema denticola*, *Prevotella intermedia*, and *Fusobacterium nucleatum*.

### Synthesis of results

A total of 62 studies were considered for summary findings, including 43 identified in the previous systematic review of studies published up to 2015 and 19 identified in the current update. Some studies reported positive associations between genotypes and detection or counts/proportions of specific bacteria, while other papers reported lack of associations (see Table 1 for details). Results divided by methods and genes are summarized below:

#### GWAS

Genome-wide significant association between host genetic variants and subgingival bacteria from participants in the Atherosclerosis Risk In Communities (ARIC) study was previously only detected at gene-centric analysis for 2 genes (KCNK1 and DAB2IP) [13, 23]. Further analysis of these data, including also a replication from an independent German sample, was carried out by using principal component analysis enriched with biologically-informed periodontal phenotypes [5]. Genome-wide significant signals were detected for associations between a series of genes and some of the identified phenotypes. Although phenotypes were identified also based on microbial colonization, no direct association between genes and bacteria were reported. Another study carried out as part of the dental ARIC study focused on a 200-kb spanning region of 1q12 revealed associations between Interferon g-inducible protein16 (*IFI16*) and absent in melanoma 2 (*AIM2*) genes and higher levels of periodontal micro-organisms [22]*.*

#### Candidate gene studies

##### Interleukin 1 genes

Positivity for ‘composite genotype’ (*IL1* +) was defined as the presence of at least one copy of ‘allele 2’ for SNPs *IL1B* rs 1,143,634 (previously reported as *IL1B* + 3953 or + 3954) and *IL1 A* rs 1,800,587 (previously reported as *IL1A* -889). The present review identified two studies on IL1 composite genotype [24, 25].

**Fig. 2:**
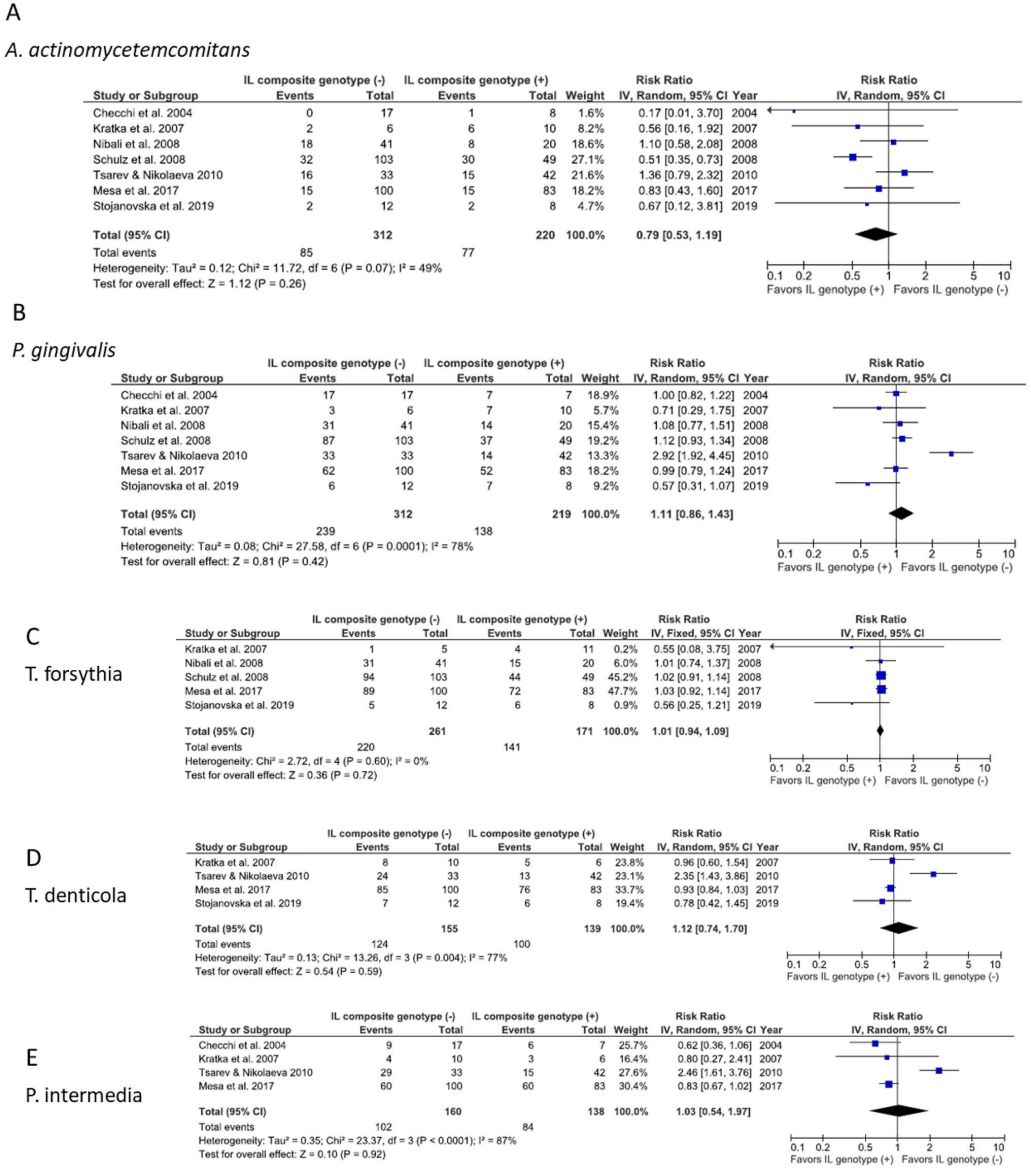
Forest plots of meta-analysis of the association between *IL1* composite genotype and detection of *A. actinomycetemcomitans* (**A**), *P. gingivalis* (**B**), *T. forsythia* (**C**), *T. denticola* (**D**) and *P. intermedia* (**E**)

Meta-analysis was conducted for association between *IL1* composite genotype and five periodontal bacteria in Caucasians, based on the 2 papers above and 5 papers identified in the previous systematic review [12, 26–29]. Forest plots of meta-analyses of the association between *IL1* composite genotype and detection of *A. actinomycetemcomitans* (Fig. 2), *P*. *gingivalis* (Fig. 2), *T*. *forsythia* (Fig. 2), *T*. *denticola *(Fig. 2) and *P*. *intermedia* (Fig. 2E) are presented in Fig. 2. The associations were not statistically significant for *A*. *actinomycetemcomitans* (overall risk ratio = 0.79, 95% CI = 0.53–1.19, *p* = 0.26, *I*^2^ test = 49%) (Fig. 2) and for *P*. *gingivalis* (overall risk ratio = 1.11, 95% CI = 0.86–1.43, *p* = 0.42, *I*^2^ = 78%) (Fig. 2). Not statistically significant associations were detected for *T*. *forsythia* (overall risk ratio = 1.01, 95% CI = 0.94–1.09, *p* = 0.72, *I*^2^ = 0%) (Fig. 2), *T*. *denticola* (overall risk ratio = 1.12, 95% CI = 0.74–1.70, *p* = 0.59, *I*^2^ = 77%) (Fig. 2), and *P*. *intermedia* (overall risk ratio = 1.03, 95% CI = 0.54–1.97, *p* = 0.92, *I*^2^ = 87%) (Fig. 2).

In a study conducted in Boston [30], the carriers of the polymorphic T allele (CT and TT genotypes) were combined into was group called *IL*-*1B*(*3954*)-*SNP* positive, while the group with the homozygous C allele (CC genotype) was named *IL*-*1B*(*3954*)-*SNP* negative. Concurrent presence of all red complex periodontal pathogens in IL-1B (3954)-SNP positive periodontitis patients was identified [30]. The frequency detection of *F*. *nucleatum* and *T*. *forsythia* was significantly higher in healthy sites in *IL*-*1B*(*3954*)-*SNP* positive compared to *IL*-*1B*(*3954*)-*SNP* negative participants. In addition, the frequency detection of *F*. *nucleatum* was found to be significantly higher in periodontitis sites in *IL*-*1B*(*3954*)-*SNP* positive compared to *IL*-*1B*(*3954*)-*SNP* negative subjects. However, due to the mixed ethnicity of the study’s participants, this study was not included in our meta-analysis.

##### Interleukin 6 gene

Some consistent associations were previously found between *IL6* -174 G (rs 1,800,795) genotypes and higher detection of *A*. *actinomycetemcomitans*, although no meta-analysis could be conducted [15]. An additional paper [31] using PCR-based methods reported lack of statistically significant associations between *IL6* SNPs and the amount of red complex species *P*. *gingivalis*, *T*. *forsythia*, and *T*. *denticola* in 326 patients with periodontitis in Italy. No meta-analysis was possible.

##### Interleukin 10 gene

Combining publications included in the previous review [15] and the current, data from six studies investigating *IL10* SNPs were available. In Asian populations with periodontitis, ATA/ATA haplotype carriers exhibited increased bacterial counts of *A*. *actinomycetemcomitans* [32]. Consistently with it, IL-10- rs1800872 AA genotype and rs1800871 TT genotypes were associated with increased *A*. *actinomycetemcomitans* counts in periodontitis [33]. No meta-analysis was possible, owing to different reporting of genetic data (in single genotypes vs. haplotypes). In Caucasians, one unspecified ‘*IL10* variant allele carrier’ showed higher *P*. *gingivalis*, *T*. *forsythia*, and *T*. *denticola* detection compared with ‘non carrier’ but no statistically significant associations [31]. In Brazilian patients with CP and periodontal health, *IL10* rs6667202 was associated with increased counts of *P*. *gingivalis* [34], while rs1800872 polymorphism was not associated with detection of studied periodontal bacteria *P*. *gingivalis*, *T*. *forsythia*, *T*. *denticola*, and *A*. *actinomycetemcomitans* [35]. In a case–control study, positive associations were found between *IL10* ATA/GCC haplotypes and the presence of *T*. *forsythia*, *Rothia dentocariosa*, *Cardiobacterium hominis*, *P*. *gingivalis*, *T*. *denticola*, *Peptostreptococcus micros*, *Synergistetes*, and *Eikenella corrodens* in subgingival samples (Inchingolo et al. 2020). Similar association was detected for GCC/GCC haplotypes with *A*. *actinomycetemcomitans* and *Porphyromonas endodontalis* [36]*.* However, the results of this study need to be considered with caution due to the risk of bias identified. Meta-analysis was not possible, due to heterogeneity of SNPs analyzed and the diversity of ethnicity in the studied populations.

##### IL-4 and IL-8 genes

In 104 patients with periodontitis, *IL4* haplotypes were significantly associated with levels of *A*. *actinomycetemcomitans* before and after periodontal treatment. On the other hand, there was no significant association between *IL8* haplotypes and subgingival levels of *A*. *actinomycetemcomitans* before and after periodontal treatment [37].

##### IL-17 A gene

*IL17A* polymorphism was significantly associated with the counts of *T*. *forsythia* and *T*. *denticola* in healthy Czech patients with periodontitis and in those with type 1 diabetes mellitus and periodontitis, respectively [20]. However, these results need to be considered with caution due to the high risk of bias identified in this study.

##### VDR gene

A cross-sectional study in 1460 Thai patients [38] revealed that *VDR*/FokI rs2228570 risk genotypes (CC + CT) were significantly associated with elevated *P*. *gingivalis* proportions and increased mean CAL. The effect of the FokI polymorphism on *P*. *gingivalis* proportions appeared greater in smokers. In another study on 326 patients with periodontitis in Italy, no significant association were reported between *VDR* gene and red complex bacteria [31].

##### Other genes

In a Brazilian study, the *NPY* polymorphism rs2521634 mutant carries proved significantly associated with subgingival *T*. *forsythia*, *Actinomyces gerencseriae*, *Fusobacterium periodonticum*, and *Prevotella nigrescens* [34]*. TBC1D1* SNP rs10010758 was associated with increased counts of *P*. *gingivalis*, while *FBX038* SNP rs10043775 proved significantly associated with decreased counts of *P*. *intermedia* [34]. In addition, no associations were identified between *CCR5Δ32* (rs333) and the presence or counts of the periodontal pathogens *P*. *gingivalis*, *T*. *forsythia*, and *T*. *denticola* in the subgingival biofilm of included patients [39]. In a study on HIV-positive North American patients with periodontitis, 8 SNPs in 6 *TLR* genes (TLR1 (*n* = 2), TLR2 (*n* = 1), TLR4 (*n* = 1), TLR6 (*n* = 1), TLR8 (*n* = 2), and TLR9 (*n* = 1)) were positively associated with *P*. *gingivalis* (2 SNPs), *T*. *denticola* (6 SNPs), and *T*. *forsythia* (1 SNP) [19]. A multi-centre study on 617 periodontitis patients with arthritis reported lack of association between the TLR4 SNP (Asp299Gly) and the presence of *P*. *gingivalis* [21].

### Risk of bias analysis

Table 2 reports results of risk of bias analysis of individual studies [17], showing a wide range of variability from a total score of 4 to a total score of 19 (out of 20) for case–control and cross-sectional studies. In addition, the only study classified as ‘longitudinal’ scored 6 (out of 8) on the Newcastle Ottawa scale [19]. The items that were lacking in most studies were representativeness of cases, power calculation, and methodological details on genetic analyses, including success rates of DNA extraction and of genotyping, good reproducibility and blind genotyping.

**Table 2:** Quality assessment of included case‐control studies with the scale proposed by Nibali et al. 2013

Author	Selection (4 items)	Comparability (1 item)	Exposure (3 items)	Study design (4 items)	Genetic analysis (8 items)
Linhartova et al. 2015	***	*	**	****	***
Cavalla et al. 2015 [14]	**	*	**	**	*****
Lauritano et al. 2016 [31]	*		*	**	
Offenbacher et al. 2016 [5]	****	*	**	**	*******
Sellers et al. 2016 [21]	****	*	***	****	*******
Mehlotra et al. 2016 [19]	***	*	**	**	*******
Linhartova et al. 2016 [20]	***		**	*	****
Mesa et al. 2017 [24]	****	*	**	**	*****
Marchesan et al. 2017 [22]	****	*	**	**	*******
Cirelli et al. 2017 [37]	***	*	**	****	*******
Cavalla et al., 2018 [34]	****	*	**	***	****
Geng et al. 2018 [32]	****	*	**	**	*****
Cavalla et al. 2018 [34]	****	*	**	***	*******
Karikova et al. 2019	****	*	**	****	*****
Stojanovska et al., 2019 [25]	**		**	**	*
Torrungruang et al. 2020 [38]	***		**	***	*****
Inchingolo et al. 2020 [36]			**	**	***
Pani et al. 2021 [30]	***		**	**	***

## Discussion

This systematic review update investigated the associations between host genetic variants and detection and counts/proportions of periodontopathogenic bacteria subgingivally, based on the concept of periodontal infectogenomics. This was defined as the effect of host genetic variants in influencing the composition of the subgingival microbiota [2]. Several new studies in this topic have been published in the last 5 years and were included in this review. The main findings could be summarized as:

- No association is seen between *IL1* composite genotype and detection of periodontopathogenic bacteria *A*. *actinomycetemcomitans*, *P*. *gingivalis*, *T*. *forsythia*, *P*. *intermedia*, and *T*. *denticola*
- Several genetic variants have been proposed as potentially having an influence on the subgingival microbiota
- When patients with periodontitis are clustered in different sub-phenotypes using microbial and inflammatory data, the association between genetic variants and disease appears to be stronger
- There is still a paucity of well-conducted studies, and in particular of studies employing -OMICS approaches in periodontal infectogenomics

Nineteen studies were included in the present review. The genetic and microbial analyses typically involved the study of one or a selected panel of SNPs and one or a selected panel of bacteria supposed to have an effect on periodontal pathology. A lack of association between *IL1* host genetic variants and subgingival periodontopathogenic bacteria had been observed in a previous systematic review [15]. Two additional studies investigating *IL1* composite genotype were identified, allowing meta-analysis of their association with five periodontopathogenic bacteria assessed by PCR from subgingival plaque samples. The absence of association with *A*. *actinomycetemcomitans*, *P*. *gingivalis*, *T*. *forsythia*, *P*. *intermedia*, and *T*. *denticola* suggests that the *IL1* composite genotype may not have any effect on influencing the composition of the subgingival microbiota, or at least not with regards to the most studied periodontopathogenic bacteria.

Among other studies with candidate-gene and candidate-bacteria approach included in this review, target SNPs were mainly within the *IL10*, *IL6*, *IL4*, *IL8*, *IL17A*, and *VDR* genes. Meta-analysis was not possible for these SNPs due to heterogeneity of SNPs analyzed and the diversity of ethnicity in the studied populations. From our analysis of these findings, some consistent associations were found for *IL10* genotypes, in Asian population and increased bacterial counts of *A*. *actinomycetemcomitans* [32] and in a Brazilian cohort, where *IL10* rs6667202 was associated with increased counts of *P*. *gingivalis* [34]. New evidence was also produced for the effect of *VDR*/FokI genotypes, which were associated with elevated *P*. *gingivalis* proportions in a Thai population [38]. *The* FokI rs2228570 CC + CT genotypes were associated with elevated *P*. *gingivalis* proportions. The effect of the FokI polymorphism on *P*. *gingivalis* proportions was greater in smokers compared to non-smokers and in alcohol drinkers compared to non-drinkers [38]. In contrast, in another study in Italy, no significant association were reported between *VDR* gene and red complex bacteria [31]. *IL4* haplotypes were associated with levels *of A*. *actinomycetemcomitans* before and after periodontal treatment [37] while *IL17A* polymorphism was associated with increased counts of *T*. *forsythia* and *T*. *denticola* in healthy Czech patients with periodontitis and in those with type 1 diabetes mellitus and periodontitis, respectively [20]. This is in line with a suspected role for these genes, involved in the host response, in disease predisposition [40].

Recent technology enabled researchers to expand this candidate-gene/candidate-bacteria approach and to perform large-scale high throughput genetic and microbiological analyses. The advantages and disadvantages of these approaches often both lie in their explorative nature which, while allowing concomitant analysis of a wide array of potentially relevant genes and bacteria, carries the risk of losing power and focus by multiple testing and by not taking into consideration a possible functional relevance to the periodontium. However, GWAS could also be interpreted with a more focused approach in the context of biological relevance. The cohort of the GWAS included in this review [5, 22] was from the Dental ARIC population, which represents one of the largest reported samples with both full-mouth periodontal clinical examinations and genotype data. The studies performed genetic and microbial analyses of 1020 White subjects participating in the ARIC and focused on 8 periodontal pathogens analyzed by checkerboard DNA-DNA hybridization. The authors hypothesized that different periodontal pathogenic pathways exist, all resulting in periodontitis. Based on principal component analysis taking into account inflammatory and microbial features, different groups of patients affected by periodontitis were identified. For example, one of the sub-phenotypes was characterized by a uniformly high pathogen load, whereas others were dominated by *A*. *actinomycetemcomitans* and *P*. *gingivalis*, respectively [5]. When patients were subdivided in these categories, genome-wide significant signals emerged with periodontal disease, which could not be detected in previous GWAS of the same population [13]. Interestingly, further analysis of the ARIC data revealed interferon g-inducible protein16 (*IFI16*) and absent in melanoma 2 (*AIM2*) genes SNPs were associated with higher levels of periodontal micro-organisms in the 1q12-locus. SNPs rs6940 and rs1057028 were significantly associated with increased *P*. *gingivalis*, *T*. *forsythia*, and *C*. *rectus* and haplotype block rs1057028 was also significantly associated with pathogens *F*. *nucleatum* and *A*. *actinomycetemcomitans* [22]. Both IFI16 and AIM2 are PYHIN inflammasome proteins that have a critical role in the innate immune response [41]. In addition, the expression of both mediators has been shown to increase in inflammatory conditions like inflammatory bowel disease [42], as well as in the inflammatory cells of the gingival tissues in patients with periodontitis [22, 43], which suggest a potential role in the response to periodontopathogenic bacteria. It was quite striking that no studies on metagenomic analysis of the subgingival microbiota were found in our search. This leaves a single study published 10 years ago and with a small sample size as the only one included in both reviews, which investigated periodontal infectogenomics with a metagenomics approach [44].

A strength of the studies in the current systematic review is the inclusion of studies carried out in several different populations and employing similar analytic strategies. It was also possible to carry out meta-analyses for *IL1* composite genotype, including a considerable number of subjects, thus increasing the sample size to assess potential genetic-microbial associations. A limitation of the included studies is their heterogeneity in data reporting and different ethnicities, as due to high variation in genotype distributions across ethnic groups, pooling data from different ethnic groups is not advisable. Moreover, three of the included studies were identified as having high risk of bias and therefore, their results should only be considered with caution. In fact, only 7 out of 19 included studies reported a priori sample size calculation for the main outcome.

Based on this review, we conclude that the *IL1* composite genotypes are not associated with specific subgingival microbial colonization patterns. We suggest that other gene variants showing promising associations with detection and counts of periodontopathogenic bacteria subgingivally, such as for example *IL10* gene variants, need replication in large independent samples. Furthermore, adherence to STREGA guidelines for the conduct and reporting of periodontal genetic-microbial association studies is of paramount importance in order to produce good-quality data [45]. Genome-wide approaches and comprehensive analyses of the microbial communities in the oral cavity, although presenting some analytical difficulties, have so far been under-performed and represent the future for research in periodontal infectogenomics.

## Supplementary Information

Below is the link to the electronic supplementary material.

Supplementary file1 (DOCX 15 KB)

Supplementary file2 (PDF 48 KB)
