# Supplementary material for: Periodontal infectogenomics: a systematic review update of associations between host genetic variants and subgingival microbial detection
Source: Clin Oral Investig. 2022 Feb 5;26(3):2209–21. doi: 10.1007/s00784-021-04233-8 (PMC8898234; doi:10.1007/s00784-021-04233-8)
Supplement: Supplementary file 1 — Supplementary file1 (DOCX 15 KB) [file 784_2021_4233_MOESM1_ESM.docx]

SUPPLEMENTAL MATERIAL 1.

PRISMA Checklist

| **Section** | # | Checklist item | Reported on page |
| --- | --- | --- | --- |
| **TITLE** | 1 | Identify the report as systematic review, meta-analysis or both | 1 |
| **ABSTRACT** | 2 | Structured summary | 2 |
| **INRODUCTION** | 3 | Rationale | 2 |
|  | 4 | Objectives | 3 |
| **METHODS** | 5 | Protocol and registration | 3 |
|  | 6 | Eligibility criteria | 3 |
|  | 7 | Information sources | 3 |
|  | 8 | Search | 4 |
|  | 9 | Study selection | 4, 5 |
|  | 10 | Data collection process | 5 |
|  | 11 | Data items | 5 |
|  | 12 | Risk of bias in individual studies | 5 |
|  | 13 | Summary measures | 5,6 |
|  | 14 | Synthesis of results | 5,6 |
|  | 15 | Risk of bias across studies | 5,6 |
|  | 16 | Additional analyses | n/a |
| **RESULTS** | 17 | Study selection | 6 |
|  | 18 | Study characteristics | 6,7 |
|  | 19 | Risk of bias within studies | 10 |
|  | 20 | Results of individual studies | 7,8,9,10 |
|  | 21 | Synthesis of results | 7 |
|  | 22 | Risk of bias across studies | 7,8,9,10 |
|  | 23 | Additional analysis | n/a |
| **DISCUSSION** | 24 | Summary of evidence | 10,11,12,13 |
|  | 25 | Limitations | 12 |
|  | 26 | Conclusions | 12,13 |
| **FUNDING** | 27 | Sources of funding | n/a |
