# Supplementary material for: Periodontal infectogenomics: a systematic review update of associations between host genetic variants and subgingival microbial detection
Source: Clin Oral Investig. 2022 Feb 5;26(3):2209–21. doi: 10.1007/s00784-021-04233-8 (PMC8898234; doi:10.1007/s00784-021-04233-8)
Supplement: Supplementary file 2 — Supplementary file2 (PDF 48 KB) [file 784_2021_4233_MOESM2_ESM.pdf]

SUPPLEMENTAL MATERIAL 2

EMBASE:

1. exp genetic predisposition/
2. exp genotype/
3. human genome/
4. exp genomics/
5. host pathogen interaction/
6. genetic variability/
7. genetic association/
8. allele/
9. exp genetic heterogeneity/
10. (single nucleotide polymorphism* or SNP* or allel* or ((gene* or geno*) adj5 (varia* or diver* or
differ*))).mp.
11. or/1-10
12. exp bacterial infection/
13. exp infection/
14. exp bacteria/
15. exp microflora/
16. dysbiosis/
17. (bacter* or microb* or pathogen* or biofilm* or microorganism* or dysbio* or (infect* adj5 agent*)).mp.
18. or/12-17
19. exp periodontal disease/
20. exp periodontics/
21. exp mouth/
22. exp dentition/
23. (subgingiva* or gingiv* or periodont* or periopathogen* or periodontopath* or ((dental or tooth or teeth or
oral*) adj5 plaque)).mp.
24. or/19-23

The above subject search was adapted for Medline, Cochrane Library and LILACS.
